# Genome-wide identification and characterization of DNA methyltransferases and demethylases in *Siraitia grosvenorii*

**DOI:** 10.3389/fpls.2025.1567781

**Published:** 2025-12-05

**Authors:** Yimei Zang, Chongnan Wang, Jiaxian Su, Lei Xie, Changming Mo, Zuliang Luo, Xiaojun Ma

**Affiliations:** 1 Biomedicine College, Beijing City University, Beijing, China; 2 State Key Laboratory for Quality Ensurance and Sustainable Use of Dao-di Herbs, Institute of Medicinal Plant Development, Chinese Academy of Medical Sciences and Peking Union Medical College, Beijing, China; 3 Guangxi Crop Genetic Improvement and Biotechnology Lab, Guangxi Academy of Agricultural Sciences, Nanning, Guangxi, China; 4 Yuelushan Laboratory, Changsha, Hunan, China

**Keywords:** Siraitia grosvenorii, C5-MTase, dMTase, fruits, gene expression

## Abstract

DNA methylation and demethylation play a crucial role in plant development, fruit ripening, and the accumulation of secondary metabolites. It is primarily catalyzed and regulated by cytosine-5 DNA methyltransferases (C5-MTases) and DNA demethylases (dMTases). In our study, six *C5-MTase* and four *dMTase* genes were identified in *Siraitia grosvenorii* genome. Phylogenetic analysis demonstrated that the six SgC5-MTase were divided into four categories, SgMET1, SgCMTs, SgDRMs, and SgDNMT2. The four SgdMTase were grouped into SgROS1, SgDML3, SgDME subfamilies. Transcript abundance levels of Sg*C5-MTase* and Sg*dMTase* genes revealed changes during vegetative and reproductive development. Furthermore, the expression of *SgdMTase* genes was upregulated during fruit ripening, while *SgCMT2/3* genes were downregulated. This indicates a potential rise in demethylation, aligning with the accumulation pattern of mogroside V. Our results suggest a role for DNA methylation modifications in the growth, development, maturation, and accumulation of mogrosides, which will also facilitate future epigenetic studies in *S. grosvenorii*.

## 1 Introduction

DNA methylation is a pivotal epigenetic modification involving the addition of a methyl group to the fifth carbon of a cytosine residue to form 5-methylcytosine (Finnegan et al., 2000; Cao et al., 2014). This modification does not alter the DNA sequence but can profoundly influence gene transcription activity by modulating chromatin architecture, recruiting specific binding proteins, or interfering with transcription factor binding. Consequently, it leads to phenotypic variation without genotypic change and serves as a core regulatory mechanism in numerous biological processes, such as regulating gene expression (Cui et al., 2016), maintaining genome stability (Li et al., 2015), facilitating genomic imprinting (Choi et al., 2002), coordinating developmental and physiological processes (Huang et al., 2019), and mediating environmental stress responses (Bharti et al., 2015). in plants.

In plants, DNA methylation occurs in the symmetrical CG and CHG (where H represents A, T, or C) contexts, as well as the asymmetrical CHH context. mCG and mCHG contexts are maintained by methyltransferase 1 (MET1) and chromomethylases 2/3 (CMT2/3), respectively, while mCHH contexts are sustained by a combination of CMT2 and domain rearranged methyltransferase 2 (DRM2) (Ma et al., 2015; Zhang et al., 2018). DRM2 is crucial for *de novo* methylation through the RNA-directed DNA methylation (RdDM) pathway, acting across both symmetric and asymmetric contexts. DNA methylation plays a critical role in regulating a wide range of biological processes. This dynamic methylation network is further precisely counterbalanced by active DNA demethylation executed by DNA demethylases (dMTases), including Demeter (DME), Repressor of Silencing 1 (ROS1), and Demeter-like Proteins 2 (DML2) and 3 (DML3) (Zhu, 2009)., together constituting a core epigenetic regulatory system that enables plants to adapt to developmental cues and environmental changes.

This sophisticated machinery is extensively involved in myriad biological processes, including leaf development, flowering time, fruit ripening, and seed development. leaf growth (Candaele et al., 2014), flowering time ( (Bai et al., 2021)), fruit ripening (Zhong et al., 2013; Liu et al., 2015), seed development (Xing et al., 2015), and hybrid vigor (Kawanabe et al., 2016). Accumulating evidence has highlighted its critical role in regulating plant secondary metabolism. DNA methylation can directly or indirectly modulate the expression of key enzyme genes in the biosynthetic pathways of important secondary metabolites—such as alkaloids, flavonoids, and terpenoids—by altering chromatin states, thereby influencing the accumulation of these high-value compounds (Zhang et al., 2018; Wei et al., 2022). For instance, in taxane biosynthesis, a CHH-type hypermethylation hotspot within the core promoter of the *BAPT* gene was identified as a Y-patch element, whose methylation level is significantly negatively correlated with taxane accumulation (Pandey and Pandey Rai, 2015).

*Siraitia grosvenorii* (monk fruit or luohanguo), a unique medicinal and edible plant indigenous to China, derives its characteristic sweetness and health benefits primarily from mogrosides, a group of triterpenoid saponins (Wu et al., 2022; Liu et al., 2018). As the most important secondary metabolites in *S. grosvenorii*, the biosynthesis and accumulation of mogrosides are undoubtedly under precise spatiotemporal control by internal developmental programs and external environmental factors. DNA methylation, acting as a bridge integrating these signals, likely plays a crucial yet unexplored regulatory role. Although the *C5-MTase* and *dMTase* gene families have been identified and preliminarily characterized in several model and crop plants, such as *Arabidopsis thaliana* (Zhang et al., 2018; Ashapkin et al., 2016), *Solanum lycopersicum* (Guo et al., 2020; Lang et al., 2017), *Citrus sinensis* (Huang et al., 2019), *Fragaria x ananassa* (Cheng et al., 2018), *Salvia miltiorrhiza* (Li et al., 2018), *Oryza sativa* (Ahmad et al., 2014), *Sorghum bicolor* (Vafadarshamasbi et al., 2022), *Zea may* (Candaele et al., 2014), and *Dendrobium officinale* (Yu et al., 2021). However, their functions in *S. grosvenorii* growth, development, and particularly in mogroside accumulation, remain entirely unknown.

Therefore, to elucidate the potential functions of DNA methylation and demethylation mechanisms in *S. grosvenorii*, this study presents the first genome-wide identification and comprehensive analysis of the *C5-MTase* and *dMTase* gene families. We systematically characterized their phylogenetic relationships, gene structures, protein-protein interaction networks, and promoter *cis*-acting elements. Furthermore, we investigated their expression profiles across various tissues (roots, stems, leaves, flowers) and during different fruit developmental stages to explore potential correlations with plant development and mogroside accumulation. Our findings provide a foundational resource for understanding the epigenetic regulation of mogroside biosynthesis and offer novel candidate genes and strategies for the epigenetic improvement of *S. grosvenorii* fruit quality.

## 2 Materials and methods

### 2.1 Plant material

*S. grosvenorii* (Cultivar Qingpiguo) were grown at the Yongfu County cultivation base (Guilin, China, GPS coordinates are E110.030835 and N24.9637). Roots, young stems, stems, young leaves, leaves, male flowers (bloom day), female flowers (bloom day), fruits at 5 days after pollination (DAP), fruits at 35 DAP, fruits at 65 DAP were harvested. Each sample contained three biological replicates, the collected samples were frozen immediately in liquid nitrogen and stored at -80 °C before use.

### 2.2 Data collection and identification of *SgC5-MTase* and *SgdMTase* genes in *S. grosvenorii*

The Arabidopsis C5-MTase protein sequence, obtained from the Phytozome database (https://phytozome.jgi.doe.gov/pz/portal.html), was used as the reference sequence. The *S. grosvenorii* genome data will be published separately. The Hidden Markov Model (HMM) for the DNA-methylase domain (PF00145) was downloaded from the Pfam database (http://pfam.xfam.org/), along with HMMs for the helix-hairpin-helix, Gly/Pro-rich loop (HhH-GPD, PF00730) and RNA recognition motif demethylase (RRM-DME, PF15628) as reference models (Yu et al., 2021). The HMMER 3.0 search tool was used to identify C5-MTase and dMTase proteins in *S. grosvenorii* (E-value ≤ 1e−10) (Wheeler and Eddy, 2013). Redundant sequences and incomplete proteins lacking key domains (PF00145, PF00730, PF15628) were manually removed. The conserved domains of SgC5-MTase and SgdMTase family members were verified using the Conserved Domain Database (NCBI-CDD, https://www.ncbi.nlm.nih.gov/cdd). The amino acid composition, molecular weight (Mw), and theoretical isoelectric point (pI) of the protein sequences were analyzed using the EXPASY tool (http://web.expasy.org/protparam). Subcellular localization of the C5-MTase and dMTase genes was predicted using Plant-mPLoc (http://www.csbio.sjtu.edu.cn/bioinf/plant-multi/).

### 2.3 Phylogenetic tree construction

To investigate the classification of C5-MTase and dMTase genes, sequences from 14 plant species were retrieved, representing various plant categories: typical dicots (*Arabidopsis thaliana* and *Solanum lycopersicum*), medicinal dicots (*Salvia miltiorrhiza*, *Ricinus communis*, and *Chrysanthemum nankingense*), typical Cucurbitaceae species (*Cucumis sativus*, *Momordica charantia*, *Cucumis melo*, *Citrullus lanatus*, and *Cucurbita moschata*), typical monocots (*Sorghum bicolor*, *Oryza sativa*, and *Zea mays*), and medicinal monocots (*Dendrobium officinale*) (**Supplementary Tables S1**, **S2**). These sequences were obtained from the NCBI Protein Database and CuGenDBv2 (http://cucurbitgenomics.org/v2/). Phylogenetic trees were constructed using Maximum Likelihood (ML) method in MEGA 11 (Yang, 2007), based on 119 aligned protein sequences with Poisson correction and 1000 bootstrap replicates. Tree visualization was performed using the Interactive Tree of Life (iTOL) online tool (Letunic and Bork, 2021).

### 2.4 Conserved motif and gene structure analysis

Conserved motifs of all *SgC5-MTase* and *SgdMTase* proteins were analyzed using the MEME Suite (v5.05) program (http://meme-suite.org) (Bailey et al., 2006). Gene structure analysis was performed using the Gene Structure Display Server (GSDS, https://gsds.gao-lab.org/) (Hu et al., 2015). The 2000-bp upstream sequences of *SgC5-MTase* and *SgdMTase* genes were extracted from the *Sg* genome, and cis-acting regulatory elements were predicted using the PlantCARE tool (http://bioinformatics.psb.ugent.be/webtools/plantcare/html/) (Lescot, 2002). Protein-protein interaction networks were constructed using the STRING 11 tool (https://string-db.org).

### 2.5 Gene expression analysis

Total RNA was extracted from three biological replicates per tissue using Ve Zol Reagent R411 (Vazyme, Beijing, China), with each replicate representing an independent plant. RNA integrity was assessed by agarose gel electrophoresis, and RNA concentration was quantified using a NanoDrop 2000C Spectrophotometer (Thermo Scientific, Waltham, MA, USA). Total RNA was reverse-transcribed into cDNA using TransScript One-step DNA Removal and cDNA Synthesis Super Mix (TransGen Biotech, Beijing, China). qRT-PCR primers (**Supplementary Table S3**) were designed using Primer Premier 6. qRT-PCR was performed in triplicate on a CFX96™ real-time PCR system (Bio-Rad, Hercules, CA, USA) using SYBR Premix Ex Taq™ (Vazyme, Beijing, China). *SgUBQ* (Shi et al., 2019) was used as the internal control, and relative mRNA levels were calculated using the 2^^−ΔΔCt^ method. Results were presented as mean ± standard deviation (SD) from three independent experiments (Livak and Schmittgen, 2001). Differential expression among tissues was analyzed by one-way ANOVA using IBM SPSS 25 software (IBM Corporation, Armonk, NY, USA).

## 3 Result

### 3.1 Identification and structural analysis of *SgC5-MTase* and *SgdMTase* genes in *S. grosvenorii*

**Figure 1:**
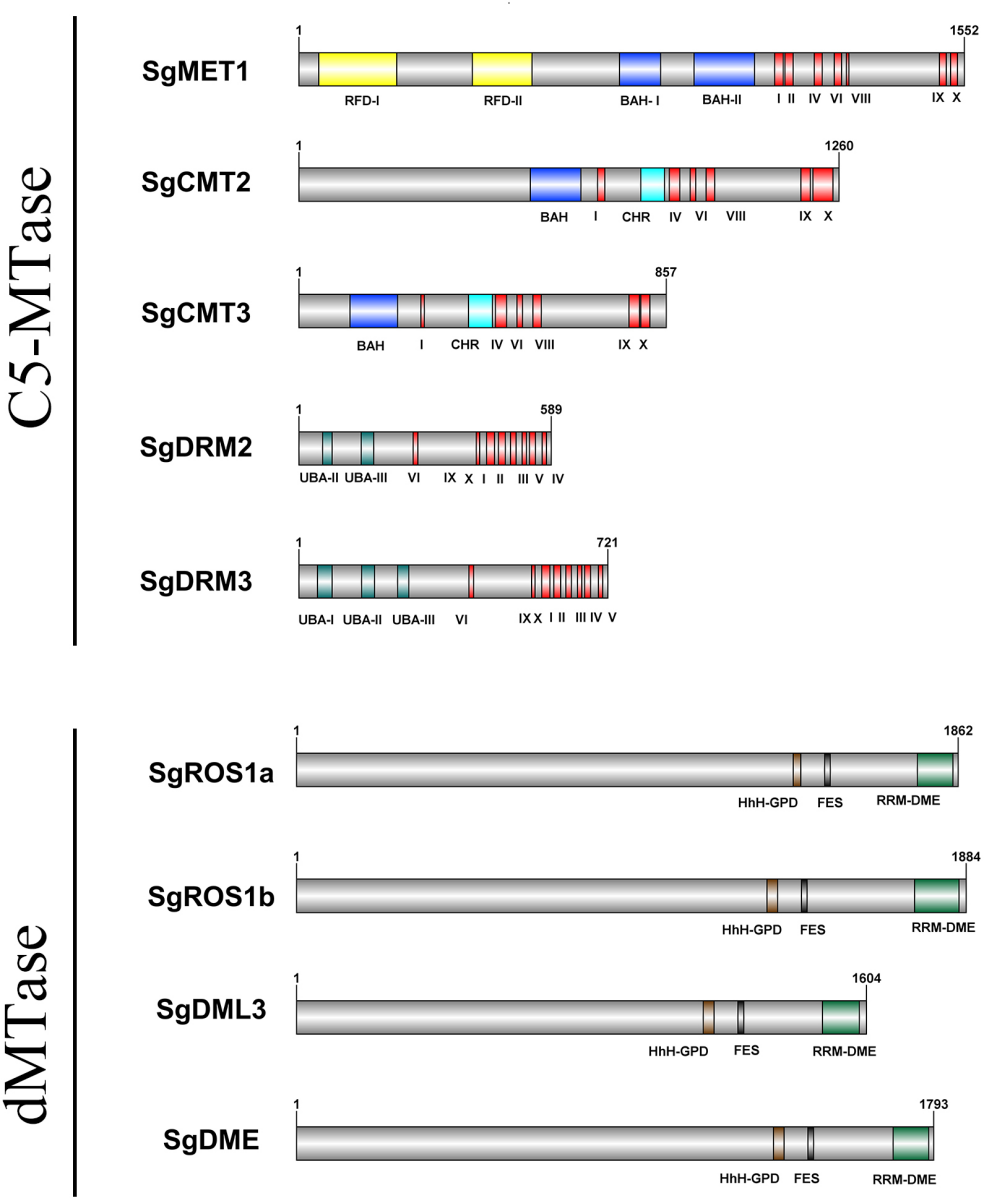
Schematic structures of SgC5-MTase and SgdMTase proteins.

A total of six *SgC5-MTase* genes were identified in the *S. grosvenorii* genome. These genes encoded proteins with conserved C-terminal catalytic domains, yet they exhibit diverse N-terminal domain combinations. Proteins containing two replication foci domains (RFD) and two bromo adjacent homology (BAH) domains at the N-terminus were classified as members of the MET family. Proteins with a BAH domain and a chromo (CHR) domain belonged to the CMT family, while those with a ubiquitin-associated (UBA) domain at the N-terminus are categorized within the DRM family. Proteins lacking N-terminal domains were classified as DNMT2 family members. Accordingly, the six genes were named *SgMET1*, *SgCMT2*, *SgCMT3*, *SgDRM2*, *SgDRM3*, and *SgDNMT2* (**Figure 1**; **Table 1**; **Supplementary Figures S1**, **S2**, **S3**, **S4**, **S1**, **S2**).

**Table 1:** Basic features of SgC5-MTases and SgdMTases.

Gene name	Gene locus	Number of amino acids	Molecular weight (kDa)	Theoretical pI	Grand average of hydropathicity (GRAVY)	Predicted subcellular localization
*SgDNMT2*	Chr02.g03017	383	44.00	6.15	0.356	Nucleus.
*SgCMT2*	Chr07.g12454	1260	142.56	8.75	-0.672	Nucleus.
*SgCMT3*	Chr08.g14718	857	97.72	5.35	-0.585	Nucleus.
*SgMET1*	Chr10.g17527	1552	175.34	8.52	-0.526	Cell membrane.
*SgDRM2*	Chr11.g19032	589	65.96	4.82	-0.407	Chloroplast.
*SgDRM3*	Chr12.g21135	720	80.58	5.45	-0.462	Chloroplast.
*SgROS1a*	Chr03.g04942	1862	208.05	5.81	-0.672	Nucleus.
*SgROS1b*	Chr04.g07782	1884	211.16	6.64	-0.7	Nucleus.
*SgDML3*	Chr06.g10888	1604	180.64	8.86	-0.702	Nucleus.
*SgDME*	Chr10.g18311	1793	201.45	6.19	-0.698	Nucleus.

Four *SgdMTase* genes were identified in *S. grosvenorii*, all of which belonged to the DME-like family. These genes encoded proteins that universally contain the HhH-GPD, FES, and RRM-DME domains at the C-terminus. Therefore, the four genes were designated as *SgDME1*, *SgDML3*, *SgROS1a*, and *SgROS1b* (**Figure 1**, **Table 1**).

The polypeptide lengths of the identified six *SgC5-MTase* genes (*SgMET1*, *SgCMT2*, *SgCMT3*, *SgDRM2*, *SgDRM3*, *SgDNMT2*) ranged from 383 to 1552 amino acids, with predicted molecular weights ranging from 44.00 to 175.34 kDa and theoretical isoelectric points (pI) from 4.82 to 8.52. While the four *SgdMTase* genes (*SgROS1a*, *SgROS1b*, *SgDML3*, *SgDME*) encoded polypeptides that range from 1664 to 1884 amino acids in length, with molecular weights between 180.64 and 211.16 kDa, and theoretical pI values ranging from 5.81 to 8.86 (**Table 1**).

### 3.2 Motif and domain analysis of SgC5-MTases and SgdMTases in *S. grosvenorii*

**Figure 2:**
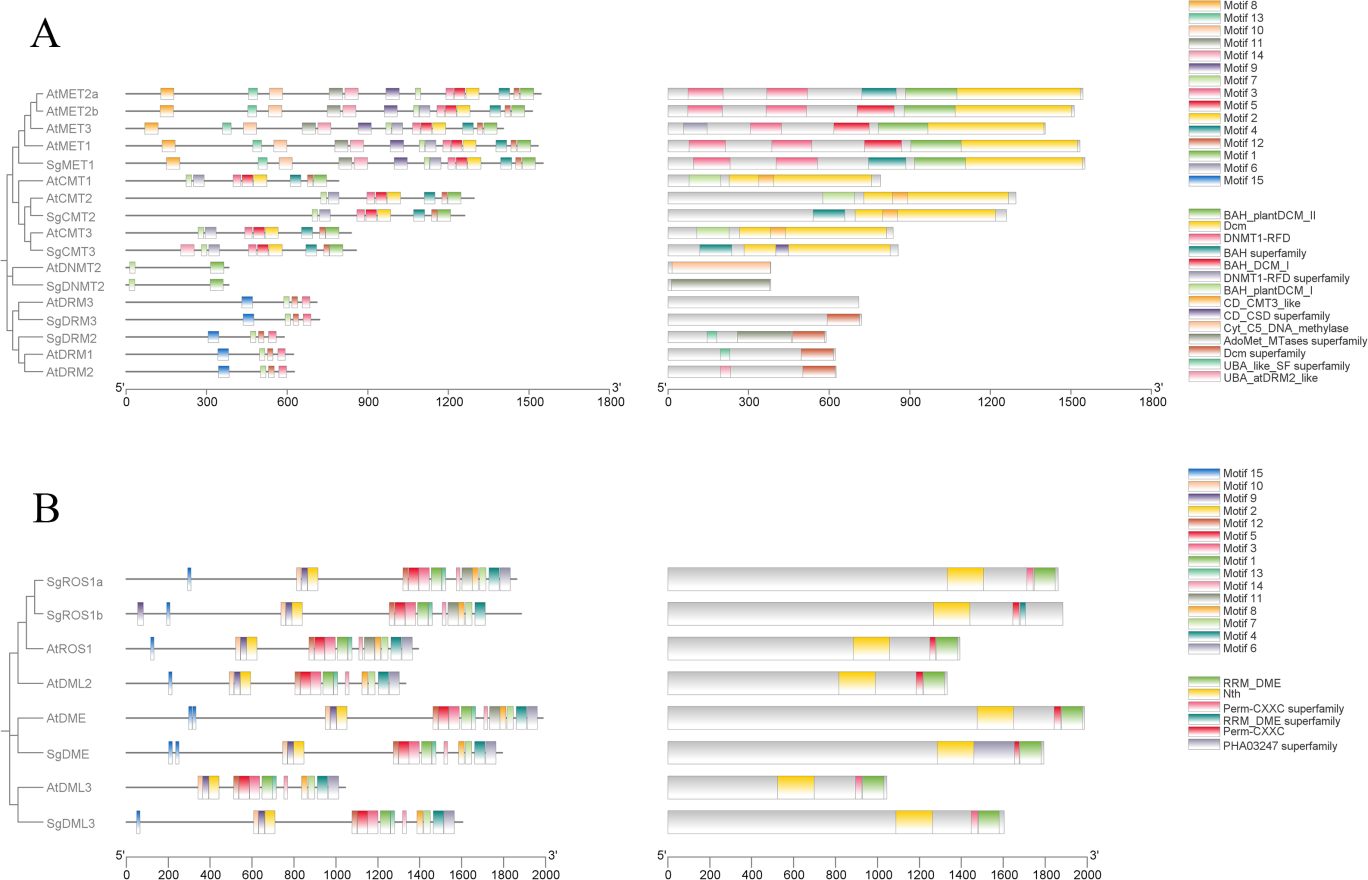
Phylogenetic relationships, conserved motifs, and gene structures of C5-MTase **(A)** and dMTase **(B)** gene families in *A thaliana* and *S. grosvenorii*.

To gain further insights into the conservation and diversity of SgC5-MTases and SgdMTases in *S. grosvenorii*, their protein motifs were examined using MEME. (E ≤ 0.01) (**Supplementary Table S4**). A total of 15 conserved motifs, ranging from 21 to 50 amino acids in length, were detected. Each C5-MTase protein contains 2 to 15 motifs. Motifs 3, 5, 7, and 15 were highly conserved in the DRM subfamily, while motifs 1 and 7 were characteristic of the DNMT2 subfamily. Motifs 1~7, 11 and 14 were predominantly located in the C-terminal region, representing the main conserved motifs in the CMT and MET subfamilies, with motif 11 within the BAH-I domain. Motifs 8, 9, 13were exclusive to the N-terminal region of the MET subfamily, with motif 8 found within the RFD-I domain, motif 13 within the RFD-II domain, and motif 9 within the BAH-II domain (**Figure 2A**).

Similarly, conserved motifs within the dMTase protein sequences of *S. grosvenorii i* and *A. thaliana* were analyzed using MEME (E ≤ 0.01) (**Supplementary Table S5**). A total of 15 conserved motifs, ranging from 17 to 50 amino acids, were identified. Each dMTase protein contains 11 to 15 motifs. Motifs 1~5, 7, 8, and 11~14 were highly conserved across all dMTases. Motifs 1, 3~8, and 11~14 were primarily located in the C-terminal region, with motifs 4 and 5 situated within the RRM-DME domain (**Figure 2B**).

### 3.3 Phylogenetic analysis of SgC5-MTases and SgdMTases in *S. grosvenorii* and other plant species

**Figure 3:**
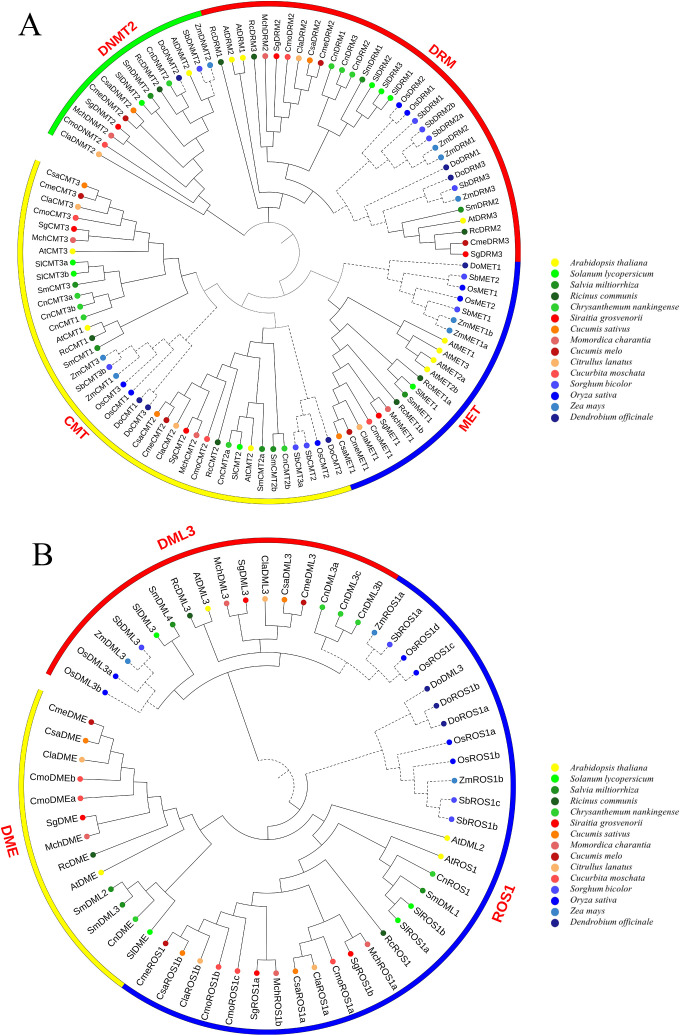
Phylogenetic analysis of the C5-MTase **(A)** and dMTase **(B)** proteins.

To investigate the evolutionary relationships of SgC5-MTase, a phylogenetic tree was constructed using 107 C5-MTase protein sequences (**Figure 3A**). The tree revealed that C5-MTase proteins from the 14 species clustered into four distinct groups: DRM, CMT, MET, and DNMT, with 33, 39, 21, and 14 members, respectively. This classification is consistent with previous studies in other plants (Qian et al., 2014; Cao et al., 2014), confirming the domain-based classification and nomenclature. The MET subfamily was further divided into dicot and monocot groups, while DRM was split into DRM2 and DRM3 clades, each further subdivided into dicot and monocot groups. Similarly, CMT was divided into CMT2 and CMT1/3 clades, with both subdivided into dicot and monocot groups. In contrast, the DNMT2 subfamily showed no clear distinction between monocots and dicots. Overall, each of the MET and DNMT2 subfamilies contained a SgC5-MTase, while the CMT and DRM subfamilies each had two SgC5-MTases. SgC5-MTases exhibited close phylogenetic relationships with C5-MTases from other Cucurbitaceae species, which is consistent with their taxonomic placement. For the dMTase proteins, 60 sequences clustered into three orthologous groups: ROS1, DML3, and DME, with 11, 21, and 28 members, respectively (**Figure 3B**). The ROS1 and DML3 groups were further divided into monocot and dicot subgroups, while the DME group was exclusive to dicots, suggesting that DME was monophyletic within dicots, consistent with prior studies. In summary, the DME and DML3 subfamilies each contained one SgdMTase, while the ROS1 subfamily included two SgdMTases. SgdMTases manifested close phylogenetic relationships with those of other Cucurbitaceae species, implying evolutionary conservation of function.

### 3.4 Predicted protein-protein interaction of SgC5-MTases and SgdMTases

**Figure 4:**
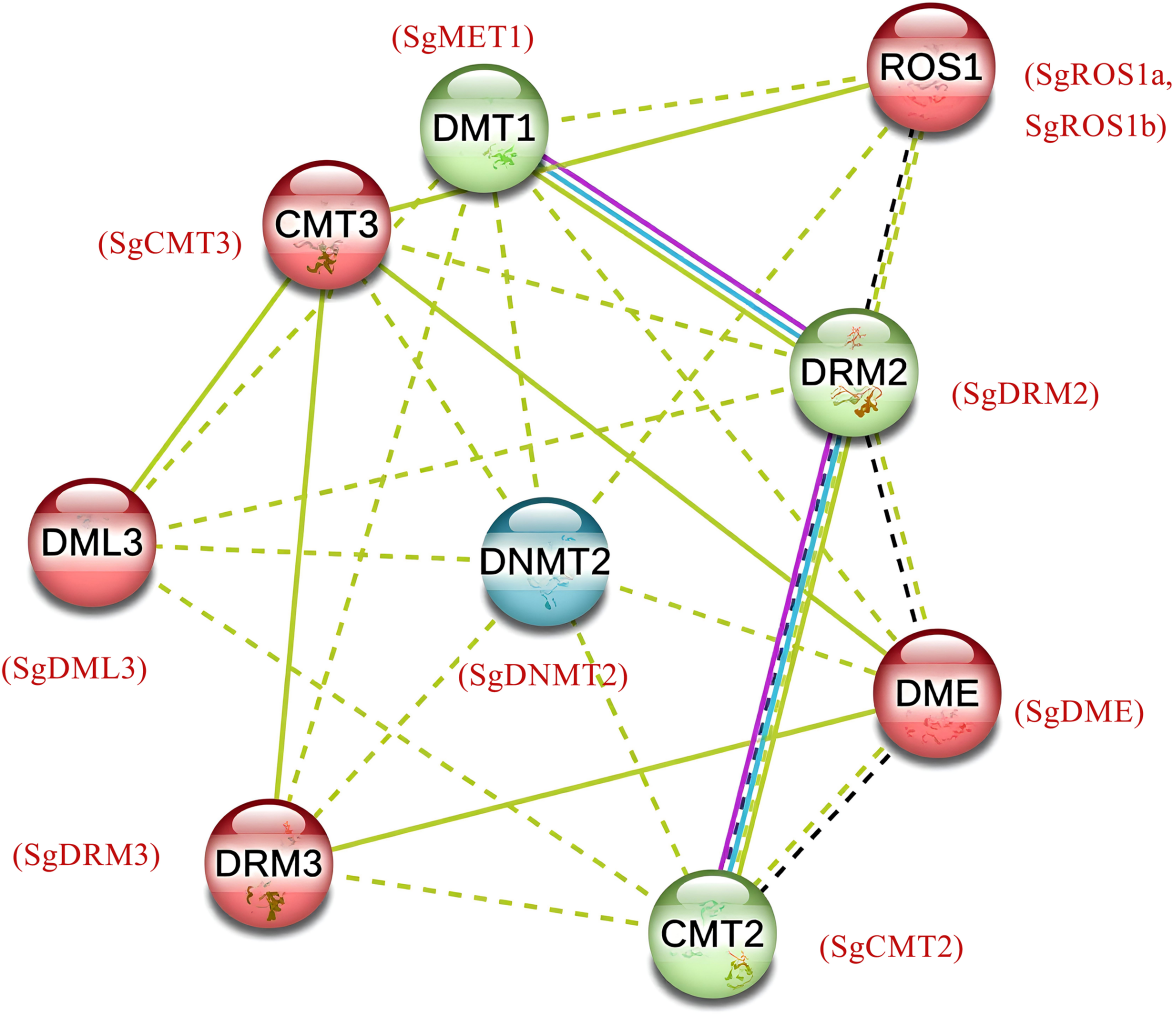
Computational prediction of protein-protein interaction network for SgC5-MTases and SgdMTases showing functional and physical associations among proteins. The dotted lines represented a relatively weak interaction while the solid lines indicated a relatively strong interaction. Colored lines between the proteins indicated the various types of interaction evidence: blue line indicated curated databases, yellow line indicated textmining evidence, black line indicated co-expression evidence and purple line indicated experimental evidence.

A protein-protein interaction network for SgC5-MTases and SgdMTases was constructed using the STRING 12 tool, based on homologous proteins from *Arabidopsis thaliana*. The PPI network revealed that SgC5-MTase and SgdMTase proteins align closely with their respective *A. thaliana* orthologs (**Figure 4**). SgDNMT2 exhibited 66.1% identity with AtDNMT2, while SgMET1 shared 59.6% identity with AtMET1. SgCMT2 and SgCMT3 were highly homologous to AtCMT2 and AtCMT3, with 59.1% and 58.2% identity, respectively. Additionally, SgDRM2 and SgDRM3 showed 56.3% and 40.3% homology with AtDRM2 and AtDRM3. SgROS1a and SgROS1b shared 44.4% and 45.4% homology with AtROS1, while SgDML3 and SgDME exhibited 49.1% and 53.8% homology with AtDME and AtDML3, respectively.

The interaction among SgMET1, SgCMT2 and SgDRM2 was observed, then SgROS1a, SgROS1b, SgDML3, SgDME and SgCMT3 were clustered, and the third cluster was formed only by SgDNMT2 (**Figure 4**). A confidence score of 0.70 for interactions between SgMET1, SgCMT2, and SgDRM2 suggested that these proteins may regulate global DNA methylation levels through protein–protein interactions or by forming complexes. SgDML3 interacted primarily with SgC5-MTases, especially SgCMTs and SgDRMs, indicating a dynamic relationship between SgC5-MTases and SgdMTases in regulating overall DNA methylation. This suggested a reciprocal negative feedback loop between SgC5-MTases and SgdMTases that modulated methylation levels.

### 3.5 Cis-acting elements analysis in *SgC5-MTase* and *SgdMTase* genes

**Figure 5:**
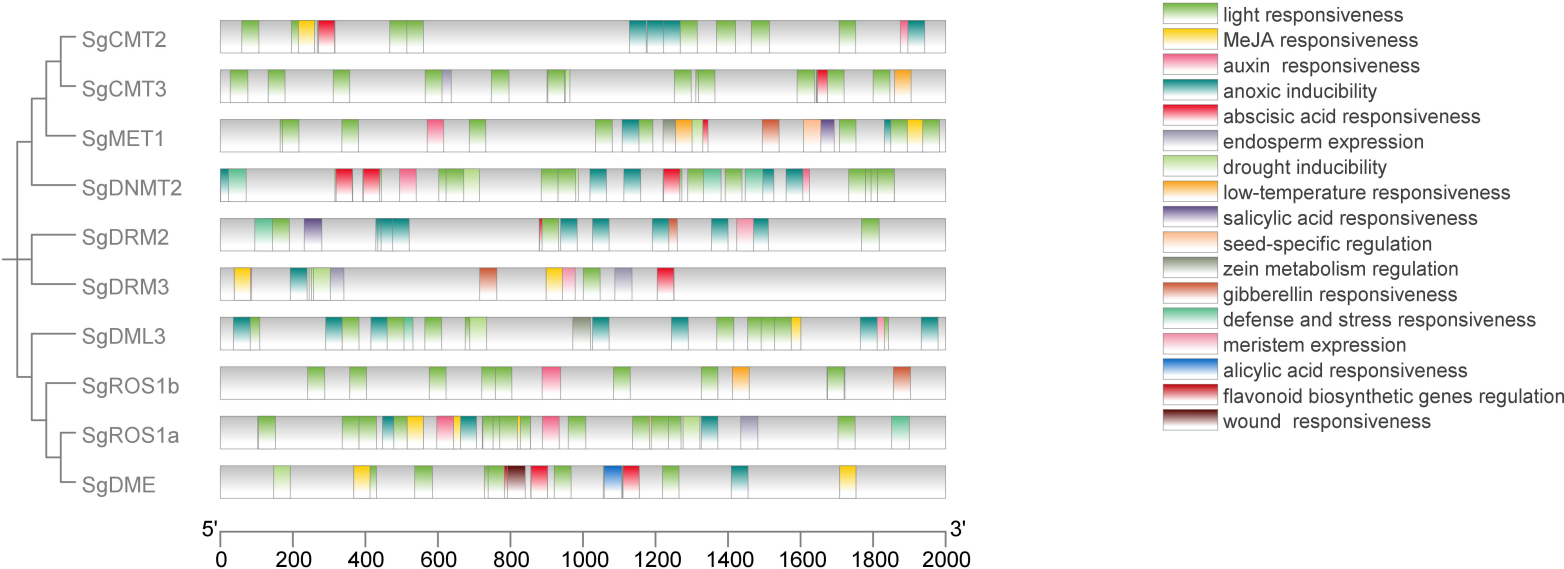
Prediction of cis-elements in the 2000-bp upstream regulatory regions of SgC5-MTase and SgdMTase genes. Different cis-responsive elements are represented by different colored boxes.

Cis-elements involved in hormone response, light response, stress response, and tissue specificity were identified in the 2000-bp upstream regulatory regions of the *SgC5-MTase* and *SgdMTase* genes using the PlantCARE database (**Figure 5**; **Supplementary Table S6**). Tissue-specific elements (6/248) were found, predominantly in the endosperm (4/6) and seed (2/6). Hormone-responsive elements (71/248) were identified, including those responsive to methyl jasmonate (MeJA) (28/71), abscisic acid (ABA) (25/71), auxin (9/71), gibberellin (GA) (5/71), and salicylic acid (SA) (4/71). Additionally, a substantial number of stress-related elements (164/248) were observed, including those responsive to anoxia (32/164), drought (10/164), low temperature (3/164), light (112/164), stress (6/164), and wounding (1/164). Furthermore, bioanabolic-responsive elements (7/248) were identified, such as those involved in alicyclic acid (1/7), meristem expression (3/7), zein metabolism regulation (2/7), and flavonoid biosynthesis regulation (1/7). These results indicated that the *SgC5-MTase* and *SgdMTase* genes may play significant roles in responding to hormones and light stress in *S. grosvenorii*.

### 3.6 Transcript abundance analysis of *SgC5-MTase* and *SgdMTase* genes

**Figure 6:**
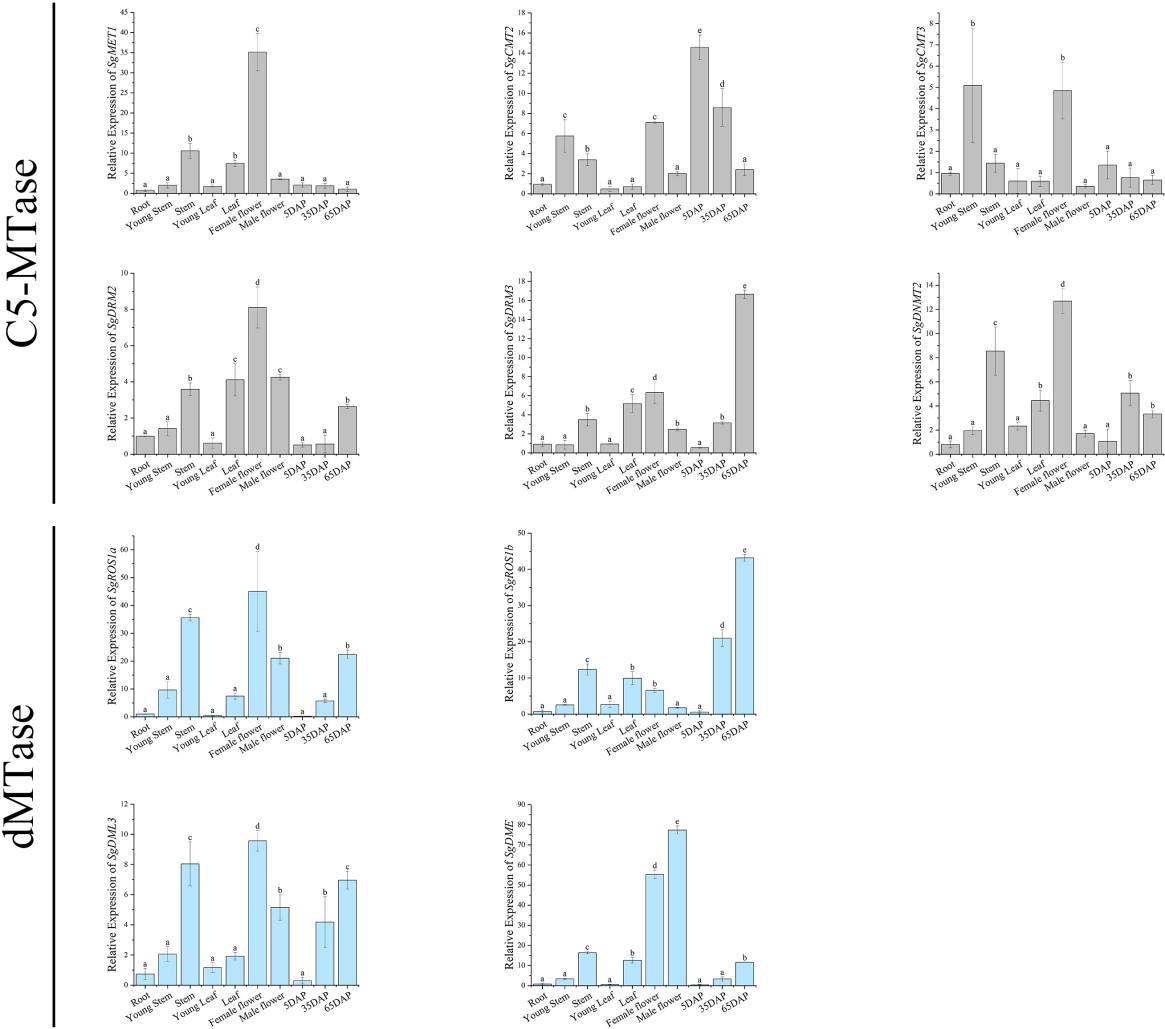
Relative expression levels of *SgC5-MTase* and *SgdMTase* genes in different tissues. (* indicates P<0.05, ** indicates P<0.01).

To explore their potential roles in plant growth and development, we analyzed the expression patterns of *SgC5-MTase* and *SgdMTase* genes across different tissues and three fruit ripening stages (**Figure 6**).

The expression of *SgMET1* was higher in stems, leaves, and female flowers, but it was relatively low in fruits. *SgCMT2/3* were highly expressed in young stems, stems, female flowers, and young fruits, with a significant down-regulation observed in later-stage fruits. In contrast, the expression of *SgDRM2/3* was more prominent in stems, leaves, flowers, and later-stage fruits than in early developmental stages, suggesting up-regulation during plant growth and fruit ripening.

The expression of all *SgdMTase* genes, similar to *DRM2/3*, was higher in mature tissues than in young tissues. SgROS1b expression was markedly elevated in late-stage fruits relative to other tissues, indicating a fruit-specific expression pattern. Additionally, *SgDME* was highly expressed in female and male flowers, approximately 55 and 77 times higher than in roots, respectively, and much higher than in other tissues. Previous studies in *A.thaliana* have shown that *DME* activated the expression of maternal FIS2, FWA, and MEA alleles, playing a key role in endosperm imprinting and seed viability (Choi et al., 2002). *DME*-mediated DNA demethylation also occurred in male gamete companion cells and coincides with the down-regulation of *DDM1* (Zhang et al., 2018). These findings suggested that *SgDME* may play a crucial role in the formation and development of male and female gametes in *S. grosvenorii*.

## 4 Discussion

*S. grosvenorii*, a perennial vine in the Cucurbitaceae family, is rich in fatty acids, essential amino acids, flavonoids, and triterpenoids. Among these, mogrosides, a class of intensely sweet non-sugar compounds of triterpenoid secondary metabolites, have significant potential for use in food additives, functional foods, and traditional Chinese medicine (Matsumoto et al., 2009). Despite its importance, the biosynthesis and regulation of mogrosides remain only partially understood, and their content is strongly influenced by environmental and developmental factors (Qiao et al., 2019). Recent studies have highlighted that epigenetic modifications, particularly DNA methylation, play pivotal roles in modulating plant development and secondary metabolism in response to external cues (Yuan et al., 2015). However, knowledge of methylation-related enzymes in *S. grosvenorii* is limited. To address this gap, we systematically identified the *SgC5-MTase* and *SgdMTase* genes across the genome, and performed an integrative analysis of their conserved motifs, phylogenetic relationships, protein-protein interactions, cis-acting elements, and transcript abundance.

DNA methylation is crucial for plant growth, with C5-MTases and dMTases playing roles in various biological processes (La et al., 2011; Ono et al., 2012). For instance, the DRM1, DRM2, CMT3 triple mutant exhibited dwarfism, partial sterility, and slow growth in *A.thaliana* (Cao and Jacobsen, 2002). C5-MTases and dMTases also significantly impact fruit ripening. Changes in the expression levels of *C5-MTase* and *dMTase* genes during the ripening process have been detected in species such as kiwifruit (Zhang et al., 2020), eggplant (Moglia et al., 2019), and grape (Shangguan et al., 2020). Active demethylation by the SlDML2 was essential for tomato fruit ripening, with loss of function mutants failing to ripen (Lang et al., 2017). Similarly, strawberry exhibited DNA hypomethylation during ripening, with *FvDRM1.3, FvDRM3.1* and genes involved in RNA-directed DNA methylation being downregulated (Cheng et al., 2018). In contrast, the process of orange fruit ripening was accompanied by a decrease in the expression of *CsdMTase* genes, correlating with increased DNA methylation levels (Huang et al., 2019). *SgC5-MTase* and *SgdMTase* genes, similar to those in *Salvia miltiorrhiza*, were generally expressed at higher levels in flowers compared to other tissues (Li et al., 2018). What’s more, *SgdMTase* genes expression increased during plant growth and fruit ripening, while *SgCMT2/3* genes expression decreased. SgMET1 expression in fruits declined, but not significantly. It was noteworthy that the expression levels of *de novo* methyltransferases *SgDRM2/SgDRM3* genes were higher in mature tissues and fruits than in young tissues and fruits, suggesting that new methylation was continuously established during plant growth and development. MET and CMT are primarily responsible for maintaining CG and CHG methylation, respectively, while DRM is involved in *de novo* methylation, mainly targeting CHH methylation (Zang et al., 2023). Our results indicated that DNA methylation in *S. grosvenorii* was a dynamic and complementary process. Our findings suggest that DNA methylation in *S. grosvenorii* is a dynamic and complementary process. Nevertheless, changes in the transcript levels of SgC5-MTases and SgdMTases across developmental stages are not sufficient to infer actual alterations in enzyme activity or the global methylation landscape. Future studies should incorporate stage-specific whole-genome bisulfite sequencing in combination with transcriptome analyses to establish the causal relationships between methylation dynamics and developmental transitions.

DNA methylation can regulate the accumulation of secondary metabolites in plants. DNA methylation modulates gene expression by influencing the binding of transcriptional activators and repressors to promoter regions of key enzymes (Elhamamsy, 2016). The expression of *phenylalanine ammonia lyase (AaPAL1)* inn *Artemisia annua*, a key enzyme in flavonoid biosynthesis, was epigenetically controlled by site-specific demethylation at transcription factor binding sites (*AaMYB1, AaMYC, AaWRKY*) in its promoter region (Pandey et al., 2019). Mogroside V, a key component of mogrosides, is a high-sweetness, low-calorie, naturally derived non-sugar sweetener that has received Generally Recognized As Safe certification from the U.S. FDA (Marone et al., 2008). Currently, it has been approved for use in over 20 countries, highlighting its promising application potential (Cui et al., 2023). The content of mogroside V remains extremely low within the first 30 DAP but increases sharply after 50 DAP (Tang et al., 2011). We noticed that the accumulation of mogroside V showed the same trend as the expression of all *SgdMTase* genes, but an opposite trend to that of the methyltransferases *SgCMT2/3* genes. The increased expression of *SgdMTase* genes and the decreased expression of *SgCMT2/3* genes may potentially reduce the methylation level in the promoter regions of key enzymes in the mogroside V synthesis pathway, facilitating the binding of transcriptional activators to these regions and thereby enhancing gene expression. To further validate these hypotheses, future studies could perform overexpression or knockout experiments of *SgC5-MTases* (e.g., *SgCMT2/3*) or *SgdMTases* (e.g., *SgROS1b*) to identify key mogroside biosynthetic genes regulated by DNA methylation. Additionally, targeted epigenetic editing approaches, such as dCas9-TET or dCas9-DNMT (Hu et al., 2021), could be employed to investigate the effects of site-specific methylation changes in candidate biosynthetic genes on both their expression and the accumulation of mogrosides. These approaches will be essential to move from correlative observations to mechanistic understanding and may provide a foundation for metabolic improvement in *S. grosvenorii*.

Evidence increasingly supports the involvement of *C5-MTase* and *dMTase* genes in abiotic stress responses (Ganguly et al., 2017; Ma et al., 2018). Cis-acting elements, as molecular switches, regulate stress-inducible gene expression and various biological processes (Yamaguchi-Shinozaki and Shinozaki, 2005). In this study, a significant number of hormone-responsive, light-responsive, and stress-responsive cis-acting elements were identified in the promoter regions of *SgC5-MTase* and *SgdMTase* genes, with notable abundance in light-responsive, hormone-responsive, and drought stress-responsive elements. Among the hormone-responsive elements identified, those responsive to MeJA were the most abundant, accounting for 39.4% of all hormone-responsive elements. Previous studies have demonstrated that MeJA treatment can significantly enhance the expression of key enzyme genes involved in mogroside biosynthesis in *S. grosvenorii* (Zhang, 2016). Based on these findings, we hypothesize that MeJA may regulate the expression of *SgC5-MTases* and *SgdMTases*, thereby influencing the methylation status of promoter regions of mogroside biosynthetic genes and ultimately modulating their transcription. Consequently, future studies could employ MeJA treatment experiments to systematically evaluate its effects on *SgC5-MTases* and *SgdMTases* expression as well as mogroside accumulation.

## 5 Conclusion

In this study, we systematically identified and characterized six SgC5-MTases and four SgdMTases in *S. grosvenorii*, and elucidated their evolutionary relationships, structural features, and expression profiles. Phylogenetic analysis categorized the six SgC5-MTases into four groups: SgCMT, SgDRM, SgMET1, and SgDNMT2, while the four SgdMTases were classified into the SgROS1, SgDML3, and SgDME subfamilies. For the first time, our results demonstrate that *SgC5-MTase* and *SgdMTase* genes display complementary expression dynamics during fruit development, which closely correspond to the accumulation pattern of mogroside V. This provides novel genome-wide evidence for a potential link between DNA methylation-related enzymes and mogroside biosynthesis. Our findings not only expand the fundamental understanding of epigenetic regulation in *S. grosvenorii*, but also lay a foundation for future studies on the molecular mechanisms by which DNA methylation regulates mogroside biosynthesis. Such insights offer potential applications in molecular breeding and the metabolic improvement of this medicinal plant.

## Data Availability

All relevant data is contained within the article: The original contributions presented in the study are included in the article/**Supplementary Material**, further inquiries can be directed to the corresponding authors.
