# Supplementary material for: Genome-wide identification and characterization of DNA methyltransferases and demethylases in Siraitia grosvenorii
Source: Front Plant Sci. 2025 Dec 5;16:1567781. doi: 10.3389/fpls.2025.1567781 (PMC12714924; doi:10.3389/fpls.2025.1567781)
Supplement: Supplementary file 1 [file DataSheet1.pdf]

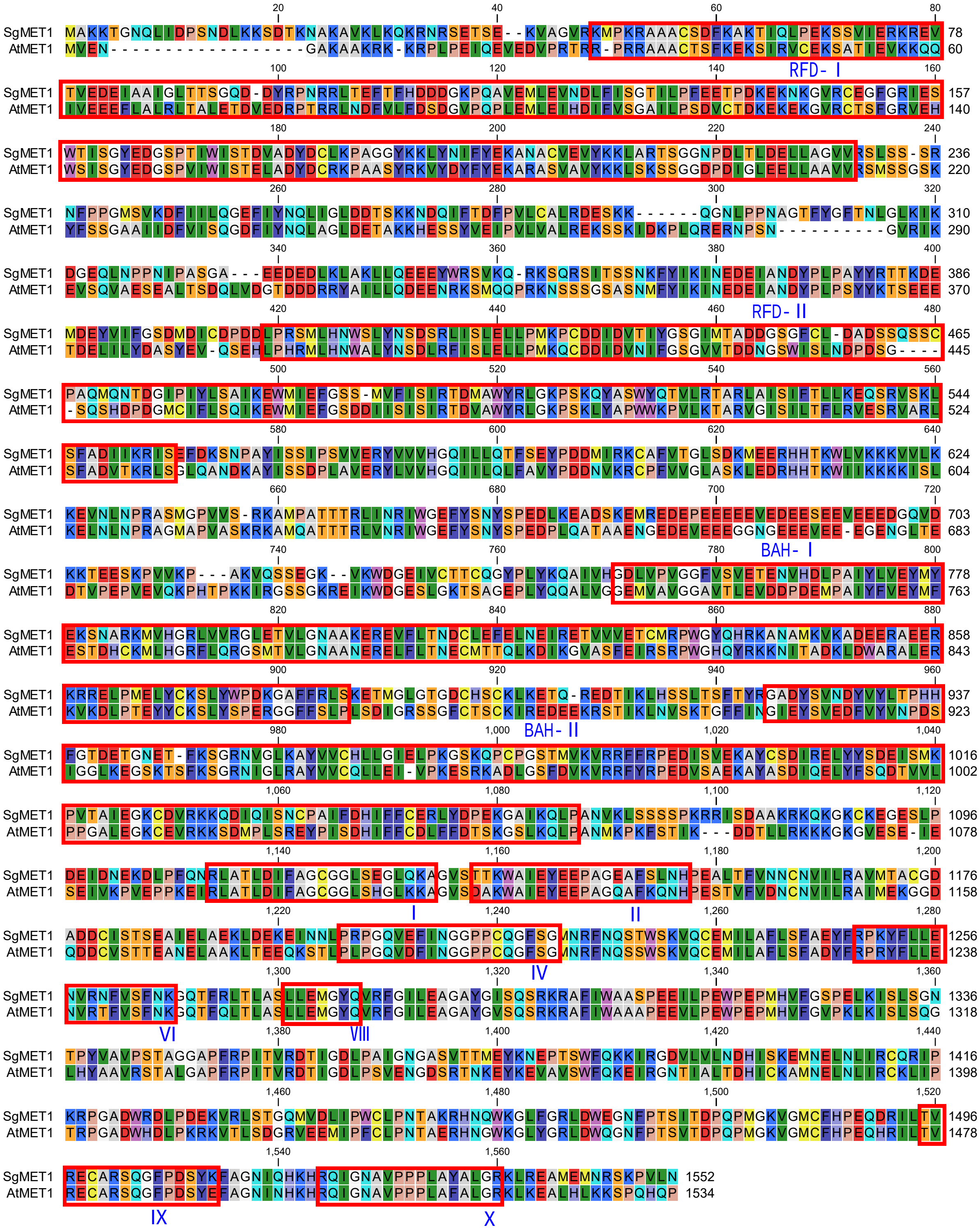

Supplemental Figure S1. Sequence alignment of MET1 protein sequences from *Siraitia grosvenorii* and *Arabidopsis thaliana*
