# Supplementary material for: Genome-wide identification and characterization of DNA methyltransferases and demethylases in Siraitia grosvenorii
Source: Front Plant Sci. 2025 Dec 5;16:1567781. doi: 10.3389/fpls.2025.1567781 (PMC12714924; doi:10.3389/fpls.2025.1567781)
Supplement: Supplementary file 7 [file DataSheet8.pdf]

```
                                20                    40                    60                    80
SgDME  MA-------- --------QT NQM------- ---------- ----SIFTNS VHTLPVSSHL E----NFGYD H--------- 30
AtDME  MNSRADPGDR YFRVPLENQT QQEFMGSWIP FTPKKPRSSL MVDERVINQD LNGFPGGEFV DRGFCNTGVD HNGVFDHGAH 80

                               100                   120                   140                   160
SgDME  --ISTTSFLI RDESSSFRK- --DGEGEFIK MFQDEAPHEH CDELLQSIVD SSCA------ GNSTPFKGMI D-----TGKQ 94
AtDME  QGVTNLSMMI NSLAGSHAQA WSNSERDLLG RSEVTSP--- ----LAPVIR NTTGNVEPVN GNFTSDVGMV NGPFTQSGTS 153

                               180                   200                   220                   240
SgDME  R----DLEID LNKTPEQRPP ---------- ---KRRQHTP VVFSGEKFTD LLNLPLDESL ---------- ------SLY- 140
AtDME  QAGYNEFELD DLLNPDQMPF SFTSLLSGGD SLFKVRQYGP PACNKPLYN- -LNSPIRREA VGSVCESSFQ YVPSTPSLFR 231

                               260                   280                   300                   320
SgDME  --EETQ--EN FVSILLDEAT QKRHDELLKD FTDTLSAAIS APCGEMKEVE KGSDQVI--- DLNKTPEQKT PKR-RKHRPK 212
AtDME  TGEKTGFLEQ IVTTTGHEIP EPKSDKSMQS IMD--SSAVN AT--EATEQN DGSRQDVLEF DLNKTPQQKP SKRKRKFMPK 307

                               340                   360                   380                   400
SgDME  VIKEGKPKRS S-KPAT-PK- ---------- ---------N TKETPSGKRK YVRKKNIKEA ATP-PVNIVE IKDLSAATKT 269
AtDME  VVVEGKPKRK PRKPAELPKV VVEGKPKRKP RKAATQEKVK SKETGSAK-- ---KKNLKES ATKKPANVGD MSNKSPEVTL 382

                               420                   440                   460                   480
SgDME  KSCRRVINFE MEKTGDLDQE KNQNE----- ---------K DMQEGTIGNF CFITRPNVSD FCTQSNGVCA TSSEVHISDW 335
AtDME  KSCRKALNFD LENPGDARQG DSESEIVQNS SGANSFSEIR DAIGGTNGSF L----DSVSQ I-DKTNGLGA MNQPLEVSMG 457

                               500                   520                   540                   560
SgDME  R-------GT MVAENVLP-- ---------- -TTQNNLARM NHM------- ---------- ----MTSFLS QP----EREA 370
AtDME  NQPDKLSTGA KLARDQQPDL LTRNQQCQFP VATQNTQFPM ENQQAWLQMK NQLIGFPFGN QQPRMTIRNQ QPCLAMGNQQ 537

                               580                   600                   620                   640
SgDME  P--------- -----EDSLN KSAYNKAEDL LNVGRILDQG KATQFQNGFS HGYTPVQQHI HAEGVEQ--- -FRNQAKRNT 432
AtDME  PMYLIGTPRP ALVSGNQQLG GPQGNKRPIF LNHQTCLPAG ------NQLY GSPTDMHQLV MSTGGQQHGL LIKNQQPGSL 611

                               660                   680                   700                   720
SgDME  Y--YKELIGL NSEYSQTVPN HPYNINEARG SKRGRPLTTQ PTQSCSINTP DSSQLCKEVL Q--TGECHRQ GSSLNVGSLE 508
AtDME  IRGQQPCVPL IDQQPAT-PK GFTHLNQMVA TSMSSPGLRP HSQSQVPTTY LHVESVSRIL NGTTGTCQRS RAPA-YDSLQ 689

                               740                   760                   780                   800
SgDME  IPGKKFESGF HATLYERYST LEANEGC--- ---SSHLNTR VCNSINSV-G FTTAMKQAM- --------LN GHIRSNQSTD 572
AtDME  ---QDIHQGN KYILSHEISN ---GNGCKKA LPQNSSLPTP IMAKLEEARG SKRQYHRAMG QTEKHDLNLA QQIAQSQDVE 763

                               820                   840                   860                   880
SgDME  RQNNWT---- KDISGDRYIN SVVHGNKFQR RQISHKLHPE IDRTCE--TT GLHKVTSYRS LIAG------ ---DKCYELQ 637
AtDME  RHNSSTCVEY LDAAKKTKIQ KVVQENL--- ----HGMPPE VIEIEDDPTD GARKGKNTAS ISKGASKGNS SPVKKTAEKE 836

                               900                   920                   940                   960
SgDME  PFPHPKAPGQ -GYTCRHSD- -----DSILK IRQAAQPIIS GSLA------ RNEVQEQGNS FGFQQFPADK TTGLLEN--- 701
AtDME  KCIVPKTPAK KGRAGRKKSV PPPAHASEIQ LWQPTPPKTP LSRSKPKGKG RKSIQDSGKA RG----PSGE L--LCQDSIA 910

                               980                 1,000                 1,020                 1,040
SgDME  EIIRKLKGLD LNDDERTTRP EQNAIVPYQG NGAVVPYVES EYVRKRKARP RVDLDPETER IWNLLMGK-E GSEGTENHEK 780
AtDME  EIIYRMQNLY LGDKER--EQ EQNAMVLYKG DGALVPY-ES ---KKRKPRP KVDIDDETTR IWNLLMGKGD EKEGDEEKDK 984

                             1,060                 1,080                 1,100                 1,120
SgDME  DKEKWWEEER KVFRGRADSF IARMHLVQGD RRFSQWKGSV VDSVIGVFLT QNVSDHLSSS AFMSLAARFP VKSISNIRTQ 860
AtDME  KKEKWWEEER RVFRGRADSF IARMHLVQGD RRFSPWKGSV VDSVIGVFLT QNVSDHLSSS AFMSLAARFP PKLSSSREDE 1064

                             1,140                 1,160                 1,180                 1,200
SgDME  GEVGMSIVTN ESAACRLYAV DSIRWDGQVL ---SLPRFAI PQTSMNHQSH RENSGSEK-N FTEVRSQIVE EEVISSQDSF 936
AtDME  RNV-RSVVVE DPEGCILNLN EIPSWQEKVQ HPSDMEVSGV DSGSKEQLRD CSNSGIERFN FLEKSIQNLE EEVLSSQDSF 1143

                             1,220                 1,240                 1,260                 1,280
SgDME  DSTITQGTGG ARSCSGSNSE AEEPIVSYNS SNIHYSNFTN IKQMETTTRS QKSFSNLNRS SVFD---EVS EHKHWQLPDG 1013
AtDME  DPAIFQSCGR VGSCSCSKSD AEFPTTRCET KTVSGTS-QS VQTGSPNLSD EICLQGNERP HLYEGSGDVQ KQETTNVAQK 1222

                             1,300                 1,320                 1,340                 1,360
SgDME  KQD-SLTSEW NEIDDLNGHS LFNLLVNIEN QQKQVPGAPS NS-QLHVTPD CGVLEVEG-- --REALSEES ISSGPSIASG 1087
AtDME  KPDLEKTMNW KD------SV CFGQPRNDTN WQT----TPS SSYEQCATRQ PHVLDIEDFG MQGEGLGYSW MSISPRVDR- 1291

                             1,380                 1,400                 1,420                 1,440
SgDME  CSTEKNMTCH SLTNG----- DLVGTLDKTS AEE---NGQA RSQETIRMEH SESVSEQSVH QQSNRIQSGS HCEYSLHLDN 1159
AtDME  -VKNKNVPRR FFRQGGSVPR EFTGQIIPST PHELPGMGLS GSSSAVQ-EH QDDTQ----H NQQDEMNKAS HLQ------- 1358

                             1,460                 1,480                 1,500                 1,520
SgDME  RDSCERNKTF PLESASITNP PQELVSPAKM QKSALSNVVH --VPAH--AE KLLDVEDRMP GKGN---QIN FSNNEVHFLS 1232
AtDME  -------KTF ----LDLLNS SEECLT---R QSSTKQNITD GCLPRDRTAE DVVDPLSNNS SLQNILVESN SSNKEQTAVE 1424

                             1,540                 1,560                 1,580                 1,600
SgDME  QADNEGNTST SKAKRRKVNS EKKSAVDWDS LRKQVESNGL IKEKSKDAMD SIDYEAIRLA SVHEISAAIK ERGMNNMLAE 1312
AtDME  Y--KETNATI LREMKGTLAD GKKPTSQWDS LRKDVEGNEG RQERNKNNMD SIDYEAIRRA SISEISEAIK ERGMNNMLAV 1502

                             1,620                 1,640                 1,660                 1,680
SgDME  RIKEFLNRLV TDHGSIDLEW LREVPPDKAK DYLLSVRGLG LKSVECVRLL TLHHLAFPVD TNVGRIAVRL GWVPLQPLPE 1392
AtDME  RIKDFLERIV KDHGGIDLEW LRESPPDKAK DYLLSIRGLG LKSVECVRLL TLHNLAFPVD TNVGRIAVRM GWVPLQPLPE 1582
                                         HhH-GPD

                             1,700                 1,720                 1,740                 1,760
SgDME  SLQLHLLELY PVLESIQKYL WPRLCKLDQR TLYELHYQLI TFGKVFCTKS KPNCNACPMR GECKHFASAF ASARLALPAP 1472
AtDME  SLQLHLLELY PVLESIQKFL WPRLCKLDQR TLYELHYQLI TFGKVFCTKS RPNCNACPMR GECRHFASAY ASARLALPAP 1662
                                                               FES

                             1,780                 1,800                 1,820                 1,840
SgDME  DEKHIVTSTN PIAAEKQPPI ----VTSPLP ILPTEASAYA ENTLGTSKCE PIIEVPATPE PEPNEITESD IEDSFY-EDP 1547
AtDME  EERSLTSATI PVPPESYPPV AIPMIELPLP LEKSLASGAP SNR---ENCE PIIEEPASPG QECTEITESD IEDAYYNEDP 1739

                             1,860                 1,880                 1,900                 1,920
SgDME  DEIPTIKLSL EEFKTTLQNY ------IPEG DMSRALVALN PEAASIPTPK LKNVSRLRTE HQVYELPDSH PLLKEMDRRE 1621
AtDME  DEIPTIKLNI EQFGMTLREH MERNMELQEG DMSKALVALH PTTTSIPTPK LKNISRLRTE HQVYELPDSH RLLDGMDKRE 1819

                             1,940                 1,960                 1,980                 2,000
SgDME  PDDPSPYLLA IWTPGETANS IQPPEQSCGS QDPDRLCNEK TCFTCNSRRE ANSQTVRGTL LIPCRTAMRG SFPLNGTYFQ 1701
AtDME  PDDPSPYLLA IWTPGETANS AQPPEQKCGG KASGKMCFDE TCSECNSLRE ANSQTVRGTL LIPCRTAMRG SFPLNGTYFQ 1899
```

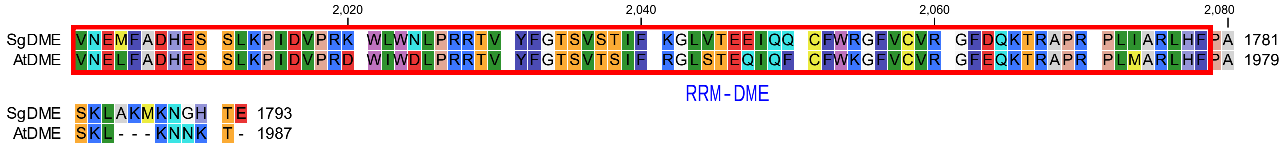

Supplemental Figure S8. Sequence alignment of DME protein sequences from *Siraitia grosvenorii* and *Arabidopsis thaliana*
